# Educational Attainment and Risk of Coronary Heart Disease Across Age Groups: Analysis of the 2021 BRFSS National Survey

**DOI:** 10.3390/healthcare14040458

**Published:** 2026-02-11

**Authors:** Ahmad Assinnari, Salman Althobaiti

**Affiliations:** 1 Department of Basic Medical Science, College of Medicine, Taibah University, Madinah 42353, Saudi Arabia; 2 Life and Health Research Centre, Taibah University, Madinah 42353, Saudi Arabia; 3 Department of Family Medicine and Community Medicine and Medical Education, College of Medicine, Taibah University, Madinah 42353, Saudi Arabia; sthobaiti@taibahu.edu.sa

**Keywords:** education, CHD, angina, myocardial infarction, risk factors

## Abstract

**Background:** The literature shows a strong association between level of education and the risk of developing Coronary Heart Disease (CHD). However, the extent to which this association attenuates after accounting for sociodemographic characteristics and cardiovascular risk factors in a survey-weighted national sample warrants further evaluation. **Objective:** We aimed to assess the association between educational attainment and angina and myocardial infarction (MI) across age groups in a nationally representative U.S. sample. **Methods:** The study analyzed 2021 Behavioral Risk Factor Surveillance System (BRFSS) data from 438,693 adults, a nationally representative telephone survey of U.S. adults. The dataset was accessed from the Centers for Disease Control and Prevention BRFSS website in February 2023. Angina and MI were identified based on self-reported physician diagnoses. Analyses included adults aged 18 years and older with no missing data for education and outcomes. **Results:** In survey-weighted analyses with college graduates as the reference group, lower educational attainment was associated with higher odds of angina and MI, compared with college graduates. In the fully adjusted model (Model 2), attending high school was associated with higher odds of angina (OR 1.439) and MI (OR 2.390). **Conclusions:** Lower educational attainment is associated with higher odds of angina and MI, particularly among younger adults. Although the magnitude of these associations was attenuated after adjustment for sociodemographic and cardiovascular risk factors, the persistence of the association underscores the importance of considering educational disparities in cardiovascular risk assessment.

## 1. Introduction

According to the Global Burden of Disease Study 2017, ischemic heart disease is the leading cause of death globally, with cardiovascular diseases together accounting for tens of millions of deaths each year, and in 2015, coronary artery disease alone accounted for 8.9 million deaths and 164 million Adjusted Life Years (DALYs) worldwide [1,2,3]. In the United States, coronary heart diseases (CHDs) are a major health concern, responsible for around 610,000 deaths each year, which accounts for approximately 1 in 4 deaths [4]. The economic impact of healthcare services for CHDs in the United States is substantial, costing over USD 200 billion each year [4,5]. Despite its significant impact on mortality and disability, angina and myocardial infarction are influenced by well-established modifiable cardiovascular risk factors, including smoking, physical inactivity, obesity, dyslipidemia, and diabetes mellitus [6].

Coronary heart disease is a complex condition influenced by various risk factors, including lifestyle, genetics, and socioeconomic factors. Several observational studies have reported that increasing attention has been directed toward understanding the relationship between sociodemographic factors and the risk of developing CHD [6,7]. Education is a key determinant of socioeconomic status (SES), and it has been widely recognized as a crucial factor in shaping health outcomes. Higher educational attainment is associated with improved access to resources, better employment opportunities, and enhanced health knowledge and behaviors. These factors, in turn, can have a significant impact on an individual’s health status, including the risk of developing CHD [8].

Several observational studies (including cohort and population-based analyses) in developed settings have reported an inverse association between educational attainment and CHD incidence or mortality [9,10,11,12,13,14,15]. Moreover, higher all-cause and cardiovascular mortality have been associated with the magnitude of lower educational attainment or less than high school education in several studies [16]. However, these studies raise concerns about causality and uncertainty regarding whether educational differences primarily reflect schooling-related social processes or correlated early-life and behavioral factors, and/or common underlying causes, such as smoking, blood pressure, obesity, and diabetes, which may confound the observed associations between education and CHD risk. Understanding the association between educational attainment and CHD is important for characterizing socioeconomic disparities in cardiovascular health. However, concerns remain regarding causality and the extent to which observed associations reflect education itself versus correlated early-life conditions or cardiovascular risk factors. Conversely, if the association is weaker or confounded by other factors, it would highlight the need to explore additional determinants of CHD risk and develop comprehensive strategies to address them [15,16,17]. However, fewer studies have examined whether educational disparities in angina and myocardial infarction vary across age groups using formal interaction testing within survey-weighted national data.

Consequently, the current study conducted a secondary analysis of a large, nationally representative survey dataset to examine the relationship between various levels of educational attainment and CHD (angina and MI) in the general adult population of the USA. The study also aimed to examine the associations between education level, angina, and MI while controlling for individual modifiable components of their risk factors among the studied subjects in different age groups. Interaction analyses were conducted to formally assess whether age modifies the association between educational attainment and angina and MI outcomes.

## 2. Materials and Methods

### 2.1. Study Population

The study included participants and their corresponding data from a large dataset acquired through the Behavioral Risk Factor Surveillance System (BRFSS), which is a comprehensive system of health-related telephone surveys that serves as the leading national data collection tool for gathering information about the health behaviors, chronic conditions, and preventive service utilization of U.S. residents. The BRFSS 2021 public-use dataset was accessed and downloaded from the CDC website in February 2023, as this was the most recent complete public-use dataset available at the time the analysis was initiated, and the manuscript was prepared. The system covers all the states, including the District of Columbia, and three U.S. territories. It involves interviewing over 400,000 adults, making it the world’s largest continuously conducted health survey system.

The BRFSS dataset is managed by the U.S. Centers for Disease Control and Prevention (CDC) and made publicly available under the CC0 1.0 Universal Public Domain Dedication license. Data are publicly available from the CDC BRFSS website (https://www.cdc.gov/brfss/annual_data/annual_2021.html (accessed on 15 February 2023)). The dataset and documentation were downloaded for secondary analysis; they do not contain any personally identifiable information. Ethical approval and informed consent were not required. Preprocessing involved importing the BRFSS dataset into Python via Google Colaboratory Environment (Google, 2023, Mountain View, CA, USA), recoding categorical variables into analytic categories, excluding respondents with missing data for education or outcome variables, and verifying consistency of variable coding prior to analysis. No imputation or data synthesis procedures were applied. The BRFSS-specific missing value codes (e.g., “Don’t know”, “Refused”) were recoded as system-missing values in SPSS; no statistical imputation was performed. For example, codes 77 or 99 were considered as “I don’t know” as they were described in the BRFSS codebook. These were set to missing and excluded listwise. All statistical analyses were subsequently performed using the Complex Samples module in SPSS to account for BRFSS survey weighting, stratification, and clustering. Participants with missing data on the primary exposure or outcome variables were excluded.

This dataset contained information on education levels and the occurrence of angina and MI among individuals belonging to various age groups as well as sociodemographic- and cardiovascular-related risk factors. More details about this large surveillance system can be found elsewhere [18].

### 2.2. Study Independent Variable

The main independent variable in this study was self-reported educational attainment, which was categorized into four levels according to the BRFSS classification: (1) did not graduate high school, (2) graduated high school, (3) attended college or technical school, and (4) graduated from college or technical school. In the regression analyses, college graduates were used as the reference category, to facilitate interpretation of excess odds associated with lower educational attainment, as it represents the highest education level.

### 2.3. Study Outcome Variables

Two outcomes of interest were assessed in this study: angina and myocardial infarction. They were selected because both represent commonly used, physician-diagnosed indicators within BRFSS surveillance. The outcome variables were obtained by asking the participant “Has a doctor, nurse or other health professional ever told you that you had angina or myocardial infarction (MI)?”. Each outcome was coded as binary (0 = no, 1 = yes) based on self-reported physician diagnosis, consistent with BRFSS definitions while not violating the international classification of diseases (ICD) [19].

### 2.4. Covariates

Additional variables considered in the study analysis included age categories (18–39, 40–49, 50–59, 60–70, and ≥ 70 years), sex (male, female), and cardiovascular risk factors. They were selected a priori based on established angina and MI risk factor frameworks and public health reports [4,5]. Cardiovascular risk factors encompassed diabetes mellitus, hypercholesterolemia, obesity (defined as a body mass index ≥ 30 kg/m^2^), current smoking status, current e-cigarette smoking, and insufficient physical activity. Physical inactivity was defined as a report of performing no physical activity or exercise over the past 30 days other than their regular job. These variables were included in adjusted models to examine the attenuation of associations after accounting for cardiovascular risk factors.

### 2.5. Ethical Consideration

This study used publicly available, de-identified survey data; therefore, Institutional Review Board (IRB) approval was not required. The protection of data privacy and confidentiality was guaranteed, with the data being exclusively utilized for research purposes.

### 2.6. Statistical Analysis

Data analysis was conducted using the Statistical Package for the Social Sciences (IBM SPSS Statistics, Version 27.0; IBM Corp., Armonk, NY, USA) using the Complex Samples module to account for the Behavioral Risk Factor Surveillance System (BRFSS) survey design, including sampling weights, strata, and primary sampling units. Categorical variables were summarized as frequencies and weighted percentages.

Associations between educational attainment and angina and myocardial infarction were examined using a sequence of survey-weighted logistic regression models. Model 0 estimated unadjusted associations between education and each outcome. Model 1 adjusted for age, sex, race/ethnicity, and marital status as potential confounders. Model 2 additionally adjusted for cardiovascular risk factors, including smoking status, diabetes mellitus, hypercholesterolemia, obesity, physical inactivity, and electronic cigarette use, recognizing these variables as potential intermediates rather than confounders. Model 1 included sociodemographic confounders, while Model 2 additionally incorporated established cardiovascular risk factors to evaluate the attenuation of associations.

To evaluate whether the association between educational attainment and angina and MI varied across age groups, interaction terms between education level and age group (education × age group) were incorporated into survey-weighted logistic regression models for both angina and myocardial infarction. Statistical significance of interaction effects was assessed using Wald F tests. All analyses accounted for the complex survey design, and statistical significance was defined as *p* < 0.05.

## 3. Results

The study analyzed data from 438,693 U.S. adults over the age of 18 years. Table 1 provides an overview of the characteristics of the studied population. More than half of the sample was aged 60 years and above (45.6%), males accounted for 46.5% of the participants, and more than half of the participants were married (52.4%). Regarding education, 6.0% had not graduated high school, 25.6% had graduated high school, 27.5% had attended college or technical school, and 40.9% had graduated from college or technical school. Among the participants, 33.5% were classified as obese, 13.0% were current cigarette smokers, and 24.4% were physically inactive.

Individuals with higher educational attainment were more likely to be female, married, not obese, less physically inactive, and less likely to smoke cigarettes or use electronic cigarettes. Additionally, the prevalence of angina and MI was progressively lower with increasing educational attainment, as shown in Table 2.

In survey-weighted unadjusted analyses (Model 0), lower educational attainment was associated with higher odds of angina and myocardial infarction compared with college graduates. After adjustment for age, sex, race/ethnicity, and marital status (Model 1), these associations remained statistically significant and were strengthened for angina, indicating confounding by sociodemographic factors.

Further adjustment for cardiovascular risk factors (Model 2) resulted in partial attenuation of the associations, although elevated odds of both angina and myocardial infarction persisted among individuals with lower educational attainment. For angina, the odds ratio comparing individuals with less than high school education to college graduates decreased from approximately 1.80 in Model 1 to 1.43 in Model 2. For myocardial infarction, the corresponding odds ratio decreased from approximately 2.93 to 2.39 after risk factor adjustment (Table 3).

Formal interaction testing demonstrated significant effect modification by age group in the association between educational attainment and angina and MI. A statistically significant education × age group interaction was observed for both angina and MI, indicating that the magnitude of educational disparities varied across age groups (Table 4).

## 4. Discussion

In this nationally representative analysis of U.S. adults, lower educational attainment was associated with higher odds of angina and myocardial infarction, even after accounting for sociodemographic characteristics and major cardiovascular risk factors. Individuals with higher education were more likely to exhibit favorable socioeconomic and lifestyle characteristics, consistent with previous studies linking educational attainment to healthier behaviors and socioeconomic advantages [20,21,22].

These findings align with a substantial body of literature reporting lower prevalence and odds of CHD among individuals with higher education and socioeconomic status [9,10,11,12,13,14,15,22,23,24]. Recent evidence has similarly demonstrated significantly lower odds of CHD among highly educated individuals [25], supporting the robustness of this association across populations and study designs.

Across sequential survey-weighted models, adjustment for sociodemographic characteristics strengthened the association between education and angina, indicating confounding by sociodemographic characteristics. Additional adjustment for cardiovascular risk factors resulted in partial attenuation of the associations for both angina and myocardial infarction, although elevated odds persisted among individuals with lower educational attainment. This pattern is consistent with prior research demonstrating close links between education and cardiovascular risk profiles, including smoking, obesity, diabetes, physical inactivity, and cholesterol levels [9,10,11,12,13,14,15]. The persistence of associations after extensive adjustment suggests that education captures broader social and contextual influences on cardiovascular health beyond individual risk factors alone.

A key contribution of this study is the demonstration of significant effect modification by age. Formal interaction testing showed that the magnitude of educational disparities in angina and myocardial infarction varied significantly across age groups, with stronger associations observed in younger and middle-aged adults and attenuation at older ages. Similar age-dependent patterns have been suggested in previous observational studies, although many relied on stratified analyses rather than formal interaction testing [21]. The attenuation at older ages may reflect selective survival, cumulative biological exposure, or diminishing marginal effects of education later in life.

Education is commonly used as a stable indicator of socioeconomic status, particularly compared with income, which may vary over time due to aging or retirement [12,13,25,26]. Other studies using alternative socioeconomic measures, such as neighborhood deprivation or life-course socioeconomic position, have also reported inverse associations with coronary heart disease incidence and mortality [6,8,9,27]. Despite differences in socioeconomic indicators and endpoints, these findings collectively underscore the importance of social determinants in cardiovascular health.

Methodologically, an important strength of this study is the application of complex sample survey methods that account for BRFSS weighting, stratification, and clustering. By using survey-weighted regression models and formal interaction testing, the analysis provides population-level estimates that more accurately reflect associations in the U.S. adult population, thereby strengthening the validity of the findings.

Several limitations should be acknowledged. First, the cross-sectional design precludes causal inference, and reverse causation cannot be excluded. Second, angina and MI were self-reported physician diagnoses, which may be subject to recall bias and non-differential misclassification and likely attenuate observed associations. Third, differential access to healthcare and diagnostic awareness by educational attainment could bias estimates in either direction. Fourth, complete-case analysis in fully adjusted models reduced the analytic sample size; while this may affect precision, the large remaining sample limits the impact on overall inference. Additionally, residual confounding by unmeasured factors, such as early-life socioeconomic conditions, cannot be ruled out [28,29]. Further, household income was not included in the analytical models due to substantial missingness in the BRFSS dataset. As a result, residual confounding by income cannot be excluded. Multiple imputation was not performed within the scope of the present analysis and represents a potential avenue for future research. Moreover, as this study is based on U.S. BRFSS data, the findings may not be directly generalizable to populations outside the United States, where educational systems, healthcare access, and socioeconomic structures differ substantially. Although the large sample size increases statistical precision, population representativeness depends on the underlying survey design and target population rather than sample size alone. Further, the narrow confidence intervals observed in this study primarily reflect the large sample size, and statistical significance should therefore be interpreted cautiously, as it does not necessarily imply large or clinically meaningful effect sizes.

In conclusion, this study confirms an inverse association between educational attainment and angina and MI in U.S. adults and demonstrates that this relationship varies across age groups. These findings highlight the importance of age-sensitive approaches and appropriate survey-weighted methods when examining socioeconomic disparities in cardiovascular health.

## 5. Conclusions

In summary, this study shows that lower educational attainment is associated with higher odds of angina and myocardial infarction among U.S. adults, even after accounting for sociodemographic characteristics and major cardiovascular risk factors. Importantly, the strength of this association is that it varies across age groups, with more pronounced educational disparities observed in younger and middle-aged adults and attenuation at older ages. These findings highlight the relevance of educational attainment as a marker of socioeconomic position in understanding population-level disparities in angina and MI. While the cross-sectional design precludes causal inference, the results underscore the importance of incorporating age-sensitive perspectives and appropriate survey-weighted methods when examining socioeconomic gradients in cardiovascular health. Future longitudinal studies are warranted to further clarify the mechanisms underlying age-dependent educational differences in coronary heart disease and inform strategies that aim to reduce socioeconomic disparities in cardiovascular health across the life course.

## Figures and Tables

**Table 1:** Characteristics of the study population (N = 438,693) *.

Characteristics		Count	%
Age groups in years	18–39	102,197	23.3%
	40–49	59,877	13.6%
	50–59	76,558	17.5%
	60–69	91,822	20.9%
	>70	108,239	24.7%
Sex	Female	234,883	53.5%
	Male	203,810	46.5%
Marital status	Not Married	206,463	47.6%
	Married	227,242	52.4%
Educational level	Not graduated high school	25,991	6.0%
	Graduated high school	111,545	25.6%
	Attended college or technical school	120,102	27.5%
	Graduated from college or technical school	178,577	40.9%
Body mass index (BMI) ≥ 30 kg/m^2^		131,305	33.5%
Physical inactivity		107,027	24.4%
Current cigarette smokers		53,832	13.0%
e-cigarette smokers		19,346	4.7%
Diabetes mellitus		61,424	14.0%
Hypercholesterolemia		149,698	40.0%
Angina		22,891	5.2%
Myocardial infarction (MI)		22,831	5.2%

* Percentages are calculated using non-missing values for each variable; therefore, denominators vary across characteristics.

**Table 2:** Subjects’ characteristics by their education level.

		Level of Education			
		Not Graduated High School	Graduated High School	Attended College or Technical School	Graduated from College or Technical School
		Count Column N %	Count Column N %	Count Column N %	Count Column N %
Sample N		25,991	111,545	120,102	178,577
Sex	Female	13,218 50.9%	56,940 51.0%	67,478 56.2%	96,075 53.8%
	Male	12,773 49.1%	54,605 49.0%	52,624 43.8%	82,502 46.2%
Marital	Not married	16,333 63.4%	61,735 55.8%	59,792 50.2%	67,853 38.4%
	Married	9428 36.6%	48,935 44.2%	59,343 49.8%	108,833 61.6%
Obesity (BMI ≥ 30), % obese		8395 38.0%	36,951 37.0%	39,915 36.9%	45,769 28.5%
Physical inactivity, % inactive		11,768 45.4%	37,828 34.0%	30,409 25.4%	26,346 14.8%
Current smoking, % current smoker		6319 26.2%	20,059 19.2%	17,231 15.1%	10,042 5.9%
Current e-cigarette use, % current user		1383 5.7%	6793 6.5%	6567 5.8%	4543 2.7%
Hypercholesterolemia, % yes		8865 44.3%	37,452 41.5%	41,501 40.6%	61,341 38.3%
Angina, % yes		1838 7.2%	6622 6.0%	6689 5.6%	7650 4.3%
MI, % yes		2402 9.4%	7287 6.6%	6723 5.6%	6304 3.5%

Denominators vary by characteristic due to item nonresponse. Values are unweighted counts and column percentages. Pearson chi-square tests (unweighted) were used to compare distributions across educational categories. Given the large sample size and the descriptive purpose of Table 2, post hoc pairwise comparisons were not performed to avoid over-interpretation of statistically significant but potentially trivial differences.

**Table 3:** Survey-weighted odds ratios (ORs) and 95% CI for angina and myocardial infarction by educational attainment (reference: college/technical school graduate).

	Complex Samples Logistic Regression * OR (CI)					
	Angina			Myocardial Infarction (MI)		
	Model (0) ^1^	Model (1) ^2^	Model (2) ^3^	Model (0) ^1^	Model (1) ^2^	Model (2) ^3^
Not graduated high school	1.643 (1.48–1.82)	1.805 (1.6–2.02)	1.439 (1.25–1.64)	2.926 (2.64–3.24)	2.930 (2.61–3.28)	2.390 (2.08–2.74)
Graduated high school	1.486 (1.37–1.60)	1.505 (1.38–1.63)	1.305 (1.18–1.44)	1.890 (1.75–2.03)	1.836 (1.70–1.97)	1.564 (1.43–1.70)
Attended college or technical school	1.449 (1.34–1.56)	1.480 (1.36–1.60)	1.313 (1.20–1.43)	1.708 (1.57–1.85)	1.703 (1.57–1.84)	1.483 (1.35–1.62)

^1^ Model 0: unadjusted. (Angina N = 432,132; myocardial infarction N = 433,736.) ^2^ Model 1: adjusted for age, sex, race/ethnicity, and marital status. (Angina N = 428,244; myocardial infarction N = 429,851.) ^3^ Model 2: additionally adjusted for smoking status, diabetes mellitus, hypercholesterolemia, obesity, physical inactivity, and electronic cigarette use. (Angina N = 321,009; myocardial infarction N = 322,403.) * All estimates are survey-weighted and account for BRFSS complex sampling design. Odds ratios greater than 1 indicate higher odds compared with college graduates.

**Table 4:** Education × age group interaction tests for angina and MI.

Outcome	Wald F	*p*-Value
Angina	161.36	<0.001
Myocardial infarction (MI)	155.94	<0.001

Interaction terms between educational attainment and age group were tested using survey-weighted logistic regression models adjusted for sex, race/ethnicity, and marital status. Statistical significance was assessed using Wald F tests.

## Data Availability

BRFSS 2021 public-use data analyzed in this study are available from the CDC BRFSS website [https://www.cdc.gov/brfss/annual_data/annual_2021.html (accessed on 15 February 2023)]. The analytic dataset was derived by the authors through the recoding and exclusion criteria described in the Methods Section.

## Abbreviations

The following abbreviations are used in this manuscript:

CHD	Coronary heart disease
DALYs	Disability-adjusted life years
SES	Socioeconomic status
BRFSS	Behavioral risk factor surveillance system
CDC	Centers for Disease Control
ICD	International classification of diseases
MI	Myocardial infarction
SPSS	Statistical package for the social sciences
